# A structural equation modeling approach to understanding pathways that connect socioeconomic status and smoking

**DOI:** 10.1371/journal.pone.0192451

**Published:** 2018-02-06

**Authors:** Sydney A. Martinez, Laura A. Beebe, David M. Thompson, Theodore L. Wagener, Deirdra R. Terrell, Janis E. Campbell

**Affiliations:** 1 Department of Biostatistics and Epidemiology, College of Public Health, University of Oklahoma Health Sciences Center Oklahoma City, Oklahoma, United States of America; 2 Department of Pediatrics, Oklahoma Tobacco Research Center, University of Oklahoma Health Sciences Center Oklahoma City, Oklahoma, United States of America; Legacy, Schroeder Institute for Tobacco Research and Policy Studies, UNITED STATES

## Abstract

The inverse association between socioeconomic status and smoking is well established, yet the mechanisms that drive this relationship are unclear. We developed and tested four theoretical models of the pathways that link socioeconomic status to current smoking prevalence using a structural equation modeling (SEM) approach. Using data from the 2013 National Health Interview Survey, we selected four indicator variables (poverty ratio, personal earnings, educational attainment, and employment status) that we hypothesize underlie a latent variable, socioeconomic status. We measured direct, indirect, and total effects of socioeconomic status on smoking on four pathways through four latent variables representing social cohesion, financial strain, sleep disturbance, and psychological distress. Results of the model indicated that the probability of being a smoker decreased by 26% of a standard deviation for every one standard deviation increase in socioeconomic status. The direct effects of socioeconomic status on smoking accounted for the majority of the total effects, but the overall model also included significant indirect effects. Of the four mediators, sleep disturbance and psychological distress had the largest total effects on current smoking. We explored the use of structural equation modeling in epidemiology to quantify effects of socioeconomic status on smoking through four social and psychological factors to identify potential targets for interventions. A better understanding of the complex relationship between socioeconomic status and smoking is critical as we continue to reduce the burden of tobacco and eliminate health disparities related to smoking.

## Introduction

An inverse association between socioeconomic status and smoking exists, although the overall mechanisms remain unclear [1–5]. The body of literature around single factors and the association with smoking is extensive, yet a comprehensive understanding of the relationship and potential mediation between multiple factors is not well understood. Researchers have attempted to test theoretical models to disentangle the pathways that link socioeconomic status to smoking, but issues with small sample sizes, a lack of generalizability, and inconsistent measurement of variables have led to inconclusive findings [6–8]. A better understanding of the pathways between socioeconomic status and smoking is crucial to identify targets for interventions that will reduce tobacco-related health disparities [9–13].

A considerable number of studies and review papers have explored individual risk factors to explain the high prevalence of smoking among populations of low socioeconomic status, such as social support, motivation, stress, psychological factors, and environmental factors.[14–16] However, most original studies use theoretical and statistical models that examine these risk factors individually rather than simultaneously, which allows consideration of both independent and dependent effects of multiple factors.[17,18] Few studies have used path analysis or structural equation modeling (SEM) to test these relationships concurrently to disentangle and separately estimate the direct and indirect pathways to smoking.[6,7,19,20]. These studies have been limited to samples of low socioeconomic status smokers to better understand the pathways to smoking cessation rather than smoking prevalence, which would be a better measure of differences in smoking uptake and cessation combined. As a statistical tool to evaluate complex relationships, SEM has the potential to significantly contribute to epidemiological studies that examine multiple factors that are often inter-related and not easily disentangled through traditional epidemiological methods.[18,21]

Our primary research objective was to develop, test, and compare alternative theoretical models of the direct and indirect pathways that connect socioeconomic status to current smoking prevalence. Using data on a large, nationally representative sample of adults from the 2013 National Health Interview Survey (NHIS), we used SEM to test our theories. The theoretical models included measures of psychological distress in order to explore whether socioeconomic factors or mental illness have a greater effect on current smoking status.

### Previous conceptual models

Many proposed conceptual models or frameworks attempt to describe potential mediators or indirect effects of socioeconomic status on smoking, such as pathways through psychosocial and environmental factors [6,7,14,22,15]. Differences in current smoking prevalence may be caused by disparities in smoking uptake or initiation and differences in smoking cessation, which are both influenced by many different factors. Moolchan et al. proposed a conceptual framework for explaining and addressing tobacco-related health disparities, which demonstrates that there have been documented disparities in initiation, patterns of tobacco use, addiction levels, access to healthcare, and success in quitting.[9] A model developed by Williams and adapted by Harwood et al. describes potential pathways from socioeconomic status to smoking, which include mediation through psychosocial pathways such as social ties, perceptions of control, stress, and affective states.[16] Businelle et al. developed and tested a conceptual model using structural equation modeling to measure the direct and indirect effects of socioeconomic status on smoking cessation through latent mediators that included social support, neighborhood disadvantage, negative affect/stress, nicotine craving, and agency. This study found all of these to be significant mediators for smoking cessation.[6]

#### Social cohesion

Neighborhood problems, such as neighborhood disadvantage, deprivation, social capital, and social cohesion, are negatively associated with socioeconomic status [23,24]. An individual’s perceived sense of social cohesion, or connectedness and trust of one’s neighborhood, has been shown to be positively associated with self-rated mental and physical health.[23] Individual ratings of neighborhood social cohesion, measured by the Social Cohesion dimension of the Collective Efficacy Scale, have been shown to be associated with lower psychological distress, measured by the Kessler K6 Scale [23,25,26]. Studies have attempted to test whether perceived social cohesion and other neighborhood contextual factors are associated with smoking prevalence or smoking cessation, but results remain inconclusive and the pathways are unclear [6,24,27,28]. Steptoe and Feldman found that smoking was not directly associated with neighborhood problems after adjusting for age, sex, and neighborhood socioeconomic status;[24] however, Alcala et al. discovered that higher social cohesion was associated with a lower likelihood of smoking among adults living with children.[29] Neighborhood problems, which include social cohesion, have been demonstrated in multiple studies to be related to psychological distress, which could mediate the relationship between socioeconomic status and smoking.[6,24,27]

#### Financial strain

Several proposed theories have hypothesized that chronic stress may account for the effects between socioeconomic status and health due to the physiological stress response.[2,14,30–33] Financial chronic stress, measured by a three-item scale that included 1) self-reported satisfaction with the current financial situation, 2) difficulty paying bills, and 3) how finances work out at the end of the month, has been demonstrated to be significantly higher among individuals in the lowest education and income categories [34]. Low socioeconomic status is known to be associated with distress, mental health issues, and poor health behaviors [30]. It is unclear whether financial strain has a role in smoking initiation, but studies have shown that smokers with high financial stress are less likely to try or successfully quit smoking and former smokers with more financial stress are more likely to relapse [35–37]. It is unclear whether the impact of financial stress on current smoking prevalence is a direct effect or if it is mediated through psychological distress or sleep disturbance.[38]

#### Sleep disturbance

The amount and quality of one’s sleep is another potential source of stress that may accumulate as part of one’s allostatic load. Researchers have proposed that sleep serves as a mediator between socioeconomic status and health [39,33]. Socioeconomic status has been demonstrated to be strongly associated with poor sleep duration, which includes both short and long durations [33,40,41]. Both income and educational attainment level are associated with poor sleep quality, which includes self-reported sleep quality as well as sleep latency (time required to fall asleep) and sleep efficiency (staying asleep) [42–44]. Sleep disturbance and sleep duration have been hypothesized to be associated with poor health behaviors, which include cigarette smoking.[33,45] Cross-sectional studies have found that cigarette smokers are significantly more likely to have poor sleep duration, report problems falling and staying asleep, and reporting daytime sleepiness compared to nonsmokers.[41,45] Longitudinal smoking cessation studies have provided evidence that sleep disturbance is significantly associated with smoking relapse after a serious quit attempt.[46,47] Optimal sleep is also significantly associated with better psychological health and fewer symptoms of depression and anxiety.[48–51] Therefore, psychological well-being could potentially mediate the relationship between sleep and smoking.

#### Psychological distress

Mental illness and psychological distress are associated with both socioeconomic status and smoking. National cross-sectional data show that as income increases, the proportion of U.S. adults with serious psychological distress decreases, with a prevalence of 8.7% among those under the federal poverty level compared to only 1.2% among adults over 400% of the poverty level.[52] The evidence is well-established that the smoking prevalence among adults with serious psychological distress or mental illness ranges between over 30% to nearly 90% depending on the condition, which is much higher than adults with no mental illness (under 20%).[53–57] Although these associations are strong and well-documented, the causal directions unclear. Most data support the theory of social causation, in which the stresses associated with low socioeconomic status leads to poor psychological health, rather than the theory of social drift in which poor mental health leads to unemployment and a movement into low socioeconomic status [58]. One study testing the effects of socioeconomic status and mental illness on smoking found that both factors have an independent association with smoking and a lower likelihood of cessation, and that the influence of mental illness was not explained by socioeconomic status.[58,59] Therefore, we hypothesized that socioeconomic status has both direct and indirect effects on smoking through psychological distress.

## Materials and methods

### Study population

We used cross-sectional data from the 2013 NHIS, which is an annual household survey representative of the resident civilian non-institutionalized U.S. population [60,61]. The NHIS is sponsored by the Centers for Disease Control and Prevention’s (CDC) National Center for Health Statistics (NCHS) and has been conducted annually since 1957.[61] The NHIS serves as the primary source for national data related to the general health of the population, and main survey topics include health indicators, health care access and utilization, and health behaviors.[62] Households were sampled using a stratified multistage sample design to gather sociodemographic and health data for households, families, and individual adults via computer assisted face-to-face interviews conducted at each household.[61] The 2013 survey included a total of 34,557 adults who completed the Sample Adult interview.

### Measures

#### Socioeconomic status

Demographic indicator variables that were part of the measurement model that underlay the latent construct of socioeconomic status included poverty ratio, personal earnings, employment status, and educational attainment. Because data on income were missing for many respondents, we used the multiply imputed values provided in publicly available NHIS imputed income files. The poverty ratio was calculated by taking the ratio of each family’s total income to the applicable Federal poverty threshold that the Census Bureau defines based on the family’s size [63]. Personal earnings represented the respondents’ best estimates of their personal earnings, including wages and tips, before taxes and deductions from all jobs in the past calendar year. Individuals who did not work in the previous year did not receive this question, and were assumed to have personal earnings of $0. Educational attainment represented the highest level of education completed and was categorized as less than 9^th^ grade, 9^th^ to 12^th^ grade no diploma, General Educational Development (GED) Diploma, high school diploma, some college, Associate’s degree, Bachelor’s degree, or a graduate degree.

#### Current cigarette smoking

Current smoking status was a single observed outcome variable in the models, and was defined using two questions: “Have you smoked at least 100 cigarettes in your entire life?” and “Do you now smoke cigarettes every day, some days, or not at all?”[64] Individuals were categorized as *never smokers* if they had not smoked 100 cigarettes in their entire life, *current smokers* if they had smoked 100 cigarettes in their entire life time and smoked every day or some days at the time of the survey [65]. *Former smokers* were defined as those who had smoked at least 100 cigarettes but were not currently smoking at the time of the survey [65].

#### Social cohesion

The NHIS survey asks respondents a series of questions about how they perceive people and relationships in their neighborhood. We used four of these questions as indicators to represent a latent variable we describe as social cohesion: people in their neighborhood help each other out, neighbors can be trusted, there are people in their neighborhood that they can count on, and their neighborhood is close-knit. For each question, respondents selected a response on a four point likert scale that reflected the extent to which they agreed with each statement (e.g. 1 = strongly disagree, 4 = strongly agree).

#### Financial strain

Seven NHIS questions related to financial strain were selected as indicators for this latent factor. The respondents reported if they were worried about not being able to pay medical costs of a serious illness or accident, being able to maintain the standard of living they enjoy, not being able to pay medical costs for normal healthcare, not having enough to pay normal monthly bills, not being able to pay rent or mortgage, or not being able to make the minimum payment on their credit cards. We scored responses to each question on a likert scale from 1 (not worried at all) to 4 (very worried).

#### Sleep disturbance

Responses to three NHIS items were used as indicators for sleep disturbance. Respondents reported the number of times in the past week that they had trouble falling asleep, that they had trouble staying asleep, and that they woke up feeling well rested.

#### Psychological distress

The NHIS includes the Kessler K6 nonspecific distress scale, a six-item assessment designed to identify individuals with serious psychological distress representing those likely to have a diagnosable mental illness [53,66]. We used these six items as indicators for a latent variable representing psychological distress. The respondents reported how often during the past 30 days they felt 1) so sad nothing could cheer them up, 2) nervous, 3) restless or fidgety, 4) hopeless, 5) worthless, and 6) that everything was an effort. The response choices to these six items were scored on a five-point likert scale and included all, most, some, a little, or none of the time with increasing values for higher distress.

### Development of four conceptual models

Using an alternative models approach to SEM, we developed four *a priori* conceptual models based on a combination of existing theories and the availability of measures in the NHIS dataset. Model 1 focuses on financial strain and social cohesion. Model 2’s pathways included sleep disturbance and psychological distress as mediators, with a direct path from socioeconomic status to smoking and an indirect path through psychological distress. Model 3 has a direct path from socioeconomic status to smoking as well as three indirect paths through each of the latent variables social cohesion, financial strain, and psychological distress. Model 4 includes all four latent mediating variables, and tests the theory that people with low socioeconomic status are subject to stressors that explain their increased smoking prevalence.

### Statistical analyses

We used SEM to evaluate our hypotheses and test whether our conceptual models were supported by the 2013 NHIS data and which model had the best fit. We developed five latent variables and tested the measurement model using confirmatory factor analysis (CFA) that specified the relationships between the observed indicators and their underlying latent constructs. In the measurement model, the latent constructs were modeled to intercorrelate freely. We conducted a square root transformation on personal earnings to stabilize its variance. Indicators for four other latent mediators (financial strain, sleep disturbance, psychological distress, social cohesion) were included in the measurement model. To accommodate the use of some categorical indicators, we estimated parameters using weighted least squares with robust standard errors (WLSMV). Parameters were therefore estimated in terms of linear regression coefficients for continuous indicators and by probit regression coefficients for categorical indicators [67].

After evaluating the fit and factor loadings of the measurement model, we specified four structural models. All four hypothesized models included a direct pathway between the latent construct, socioeconomic status, and an observed current smoking status, but differed in the number and type of indirect pathways through the other latent constructs. Parameter estimates were obtained using the weighted least squares estimators and standard errors for the indirect effects were estimated using the theta method. Goodness of fit indices included the comparative fit index (CFI), Tucker-Lewis Index (TLI), and the root-mean-square error of approximation (RMSEA), and we considered a fit of >0.95 for the CFI and TLI and < 0.06 for RMSEA to indicate adequate fit.[68] We did not use the chi-square goodness of fit test or the weighted root-mean-square residual because these indices are not informative with very large sample sizes [69]. We evaluated model fit statistics using published recommendations for significance [70,68,71]. Missing data were handled by pairwise deletion, which treats missingness as a function of the observed covariates but not of the observed outcomes [67,72].

We used the Mplus software package, version 7.4 for all modeling, SAS version 9.4 (SAS Institute, Cary, NC) for all data cleaning and recodes, and SAS-Callable SUDAAN version 11 (Research Triangle Institute, Research Triangle Park, NC) for descriptive analyses [67,73,74]. We computed standard errors and model fit statistics taking into account stratification, non-independence due to cluster sampling, and unequal probability of selection that are features of complex survey data. We did this by using the TYPE = COMPLEX option in the ANALYSIS command of Mplus and specified the strata, cluster, and weight variables provided in the NHIS data. We used the TYPE = IMPUTATION command to combine the estimates and standard errors from analyses of the five multiply imputed NHIS data files [67].

## Results

### Demographic characteristics and behaviors

**Table 1**displays the weighted percentages and 95% Confidence Intervals (95% CI) of demographic characteristics and behaviors of U.S. adults based on the 2013 NHIS survey that consisted of 34,557 adult respondents. The population had a weighted average age of 46.8 years and consisted of nearly 52% females, 80% whites, and 12% blacks. The prevalence of current smoking was estimated to be 17.8%, while an additional 21.9% were former smokers. **Table 1**shows that less than 5% of data were missing for the majority of variables. The only variable with a high proportion of missing data was financial worry related to paying for credit cards, but this was because the question did not apply to many individuals who did not have a credit card.

**Table 1:** Demographic characteristics and behaviors.

Characteristic	Weighted Mean (SD) / Weighted Percentage (95% CI)	Percentage missing
**Demographics**		
Age (years)	46.8 (0.16)	0
Gender (% female)	51.8 (51.1, 52.6)	0
Race		0
(% white)	79.8 (79.1, 80.5)	
(% black)	12.0 (11.5, 12.6)	
(% Asian)	5.6 (5.2, 5.9)	
(% other)	2.4 (2.1, 2.7)	
Ethnicity (% Hispanic)	15.0 (14.4, 15.7)	0
**Smoking status**		0.4
(% current)	17.8 (17.2, 18.4)	
(% former)	21.9 (21.3, 22.6)	
(% never)	60.0 (59.4, 61.0)	
**Socioeconomic status**		
Poverty ratio	3.78 (0.03)	0
Personal earnings (dollars)	26,523 (288)	0
Education		0.5
(% less than 9^th^ grade)	4.7 (4.3, 5.0)	
(% 12^th^ grade no diploma)	9.1 (8.7, 9.5)	
(% GED)	2.9 (2.7, 3.2)	
(% high school diploma)	23.1 (22.5, 23.8)	
(% some college)	19.8 (19.3, 20.5)	
(% Associate’s degree)	10.9 (10.5, 11.4)	
(% Bachelor’s degree)	19.1 (18.6, 19.8)	
(% Graduate degree)	10.0 (9.6, 10.6)	
Employment status (% unemployed)	34.3 (33.0, 34.5)	0
**Financial strain (worried about money for…)**		
Retirement (1 to 4 scale)	2.57 (0.009)	3
Medical costs for illness (1 to 4 scale)	2.61 (0.010)	3
Maintaining standard of living (1 to 4 scale)	2.70 (0.008)	3
Medical costs for normal healthcare (1 to 4 scale)	2.90 (0.009)	3
Normal monthly bills (1 to 4 scale)	2.95 (0.008)	3
Rent, mortgage, or housing costs (1 to 4 scale)	3.12 (0.009)	3
Credit cards (1 to 4 scale)	3.32 (0.010)	35
**Sleep disturbance**		
Difficulty falling asleep (number of times in past week)	1.30 (0.018)	3
Difficulty staying asleep (number of times in past week)	1.64 (0.022)	3
Not feeling well rested after waking (number of days in past week)	2.70 (0.024)	4
**Psychological distress**		
So sad nothing could cheer you up (1 to 5 scale)	0.42 (0.006)	3
Nervous (1 to 5 scale)	0.62 (0.008)	3
Restless or fidgety (1 to 5 scale)	0.64 (0.009)	4
Hopeless (1 to 5 scale)	0.25 (0.005)	4
Everything was an effort (1 to 5 scale)	0.55 (0.009)	4
Worthless (1 to 5 scale)	0.19 (0.005)	4
**Social cohesion**		
Neighbors help each other out (1 to 4 scale)	1.18 (0.003)	6
There are neighbors I can count on (1 to 4 scale)	1.19 (0.003)	5
Neighbors can be trusted (1 to 4 scale)	1.18 (0.003)	6
Close-knit neighborhood (1 to 4 scale)	1.35 (0.004)	6

### Correlations

**Fig 1:**
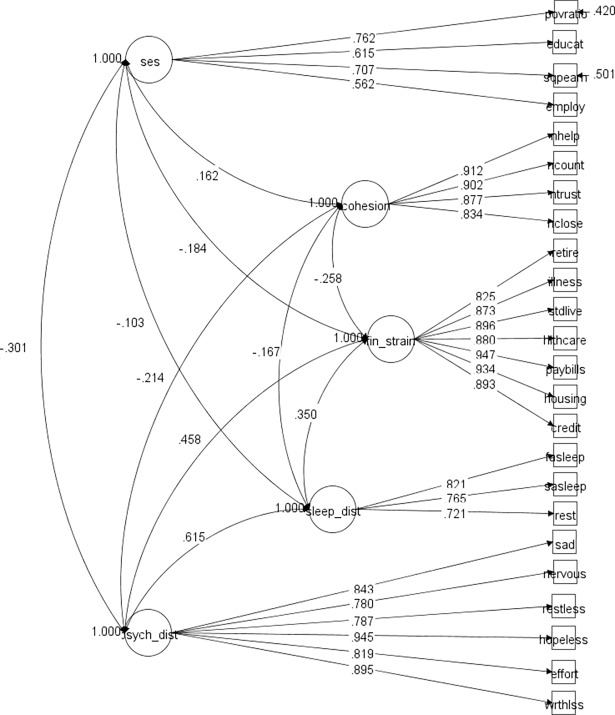
Confirmatory factor analysis–measurement model standardized results.

Our CFA, which was conducted to assess the adequacy of the hypothesized measurement model, consisted of 5 latent variables and 24 manifest variables (**Fig 1**). The results of the CFA indicated that the hypothesized measurement model fit the data adequately, with a RMSEA of 0.052, a CFI of 0.968, and a TLI of 0.963. The standardized factor loadings for all five latent variables were statistically significant and above 0.56 and most were above 0.70. The magnitude and significance of the factor loadings suggest that all indicators were moderately or strongly correlated with the latent factor with which they were hypothesized to be related. We examined the interrelationships among the latent constructs and found that the socioeconomic status construct was inversely correlated with financial strain, sleep disturbance, and psychological distress and positively correlated with social cohesion. The largest correlation was between socioeconomic status and psychological distress (-0.301). We also examined the zero-order correlations between all observed variables (**Table 2**). The highest correlations were found between indicators within the same latent construct. Only weak associations were found between observed variables that were linked to different constructs. Based on these results, we retained the proposed measurement model without any modifications.

**Table 2:** Zero-order correlations.

**Variable**	**1**	**2**	**3**	**4**	**5**	**6**	**7**	**8**	**9**	**10**	**11**	**12**	**13**
**Socioeconomic status**													
**1**. Poverty ratio	-												
**2**. Personal earnings	0.510	-											
**3**. Education	0.462	0.373	-										
**4**. Employment	0.305	0.946	0.289	-									
**Financial strain**													
**5**. Retirement	-0.183	0.072	-0.090	0.186	-								
**6**. Medical costs, illness	-0.244	0.000	-0.153	0.146	0.771	-							
**7**. Maintain std of living	-0.223	-0.009	-0.134	0.098	0.795	0.808	-						
**8**. Normal healthcare	-0.284	-0.050	-0.196	0.088	0.718	0.853	0.819	-					
**9**. Normal monthly bills	-0.355	-0.093	-0.219	0.052	0.710	0.723	0.817	0.786	-				
**10**. Rent, mortgage	-0.322	-0.059	-0.195	0.070	0.679	0.691	0.778	0.753	0.919	-			
**11**. Credit cards	-0.300	-0.053	-0.196	0.093	0.665	0.679	0.732	0.740	0.870	0.874	-		
**Sleep disturbance**													
**12**. Falling asleep	-0.113	-0.124	-0.067	-0.114	0.247	0.216	0.258	0.215	0.254	0.236	0.210	-	
**13**. Staying asleep	-0.023	-0.070	-0.002	-0.114	0.247	0.204	0.245	0.186	0.214	0.187	0.156	0.686	-
**14**. Feeling rested	-0.073	0.010	-0.024	0.037	0.293	0.254	0.292	0.232	0.280	0.267	0.246	0.504	0.529
**Psychological distress**													
**15**. Sad	-0.261	-0.228	-0.193	-0.199	0.326	0.309	0.377	0.328	0.407	0.380	0.365	0.403	0.348
**16**. Nervous	-0.112	-0.098	-0.022	-0.076	0.310	0.281	0.332	0.251	0.324	0.288	0.283	0.408	0.356
**17**. Restless/fidgety	-0.115	-0.088	-0.066	-0.075	0.294	0.266	0.321	0.253	0.316	0.280	0.267	0.484	0.450
**18**. Hopeless	-0.300	-0.233	-0.199	-0.198	0.400	0.359	0.454	0.386	0.483	0.441	0.447	0.432	0.372
**19**. Effort	-0.203	-0.167	-0.122	-0.153	0.319	0.301	0.359	0.301	0.385	0.347	0.337	0.403	0.377
**20**. Worthless	-0.289	-0.270	-0.200	-0.242	0.346	0.317	0.393	0.334	0.427	0.383	0.399	0.405	0.353
**Social cohesion**													
**21**. Help	0.177	0.054	0.130	-0.019	-0.173	-0.188	-0.204	-0.191	-0.225	-0.215	-0.199	-0.132	-0.078
**22**. Count on	0.200	0.043	0.137	-0.045	-0.178	-0.197	-0.209	-0.218	-0.245	-0.241	-0.233	-0.116	-0.058
**23**. Trust	0.280	0.088	0.202	-0.017	-0.192	-0.216	-0.226	-0.238	-0.285	-0.276	-0.278	-0.141	-0.063
**24**. Close knit	0.105	0.022	0.048	-0.040	-0.144	-0.150	-0.162	-0.134	-0.163	-0.152	-0.128	-0.125	-0.096
**25. Smoking**	-0.303	-0.059	-0.257	0.089	0.183	0.197	0.179	0.201	0.252	0.230	0.220	0.163	0.026
	**14**	**15**	**16**	**17**	**18**	**19**	**20**	**21**	**22**	**23**	**24**	**25**	
**Socioeconomic status**													
1. Poverty ratio													
2. Personal earnings													
3. Education													
4. Employment													
**Financial strain**													
5. Retirement													
6. Medical costs, illness													
7. Maintain std of living													
8. Normal healthcare													
9. Normal monthly bills													
10. Rent, mortgage													
11. Credit cards													
**Sleep disturbance**													
12. Falling asleep													
13. Staying asleep													
14. Feeling rested	-												
**Psychological distress**													
15. Sad	0.342	-											
16. Nervous	0.357	0.634	-										
17. Restless/fidgety	0.420	0.604	0.725	-									
18. Hopeless	0.384	0.815	0.682	0.659	-								
19. Effort	0.405	0.689	0.643	0.645	0.751	-							
20. Worthless	0.373	0.766	0.639	0.638	0.868	0.748	-						
**Social cohesion**													
21. Help	-0.135	-0.187	-0.129	-0.146	-0.199	-0.169	-0.181	-					
22. Count on	-0.129	-0.185	-0.100	-0.121	-0.202	-0.153	-0.183	-0.830	-				
23. Trust	-0.147	-0.199	-0.110	-0.147	-0.215	-0.180	-0.188	-0.766	-0.791	-			
24. Close knit	-0.142	-0.146	-0.127	-0.139	-0.155	-0.149	-0.137	-0.781	-0.736	-0.742	-		
**25. Smoking**	0.141	0.203	0.172	0.187	0.217	0.189	0.203	-0.138	-0.179	-0.227	-0.105	-	

### Results from four alternative SEMs

Model fit statistics are summarized for all four models in **Table 3**. The first model, which posited strain and social cohesion as mediators of the effects of socioeconomic status on current smoking, fit poorly (RMSEA = 0.81). The second model with mediation through sleep disturbance and psychological distress had poor fit based on values of CFI (0.934) and TLI (0.917) less than 0.95. The third model allowed mediation through social cohesion, financial strain, and psychological distress. This model showed acceptable fit based on values of CFI and TLI, but the RMSEA (0.62) was higher than desired for adequate fit. The fourth model, which permitted the effects of socioeconomic status to be mediated through all four factors (social cohesion, financial strain, sleep disturbance, and psychological distress) had adequate fit based on all three fit indices including the RMSEA (0.055), CFI (0.960), and TLI (0.955).

**Table 3:** Fit statistics from four alternative models for socioeconomic status to current smoking status (3-levels).

	RMSEA	CFI	TLI
Model 1: Social Cohesion, financial strain	0.081	0.965	0.958
Model 2: Psychological distress sleep disturbance	0.055	0.934	0.917
Model 3: Social Cohesion, financial strain, psychological distress	0.062	0.961	0.956
Model 4: Social Cohesion, financial strain, psychological distress, sleep disturbance	0.055	0.960	0.955

**Fig 2:**
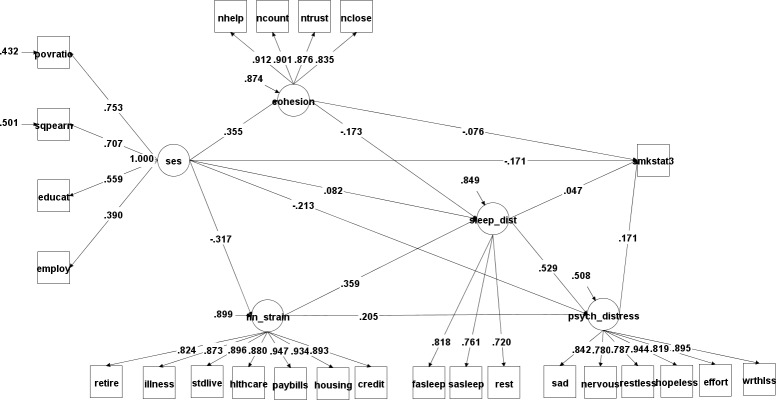
Structural equation model 4 standardized results*–pathways to current smoking status (3-levels) through financial strain, social cohesion, psychological distress, and sleep disturbance.

Model 4 (Fig 2) generated the best model fit statistics. **Table 4**presents a decomposition of the standardized direct, indirect, and total effects of socioeconomic status on current smoking prevalence as well as the specific indirect effects through various pathways and the total effects of each of the model’s mediating variables. In the fourth model, which includes mediating pathways through all four latent variables, socioeconomic status had a significant direct (-0.171), indirect (-0.087), and total (-0.258) effects on current smoking. The model infers that the probability of being a smoker decreased by 26% of a standard deviation for every one standard deviation increase in socioeconomic status. About two-thirds of this total effect was a direct result of socioeconomic status and the other third was indirect, mediated through four latent constructs. Higher socioeconomic status was associated with greater social cohesion, which was associated with a lower smoking prevalence. Higher socioeconomic status decreased financial strain and a higher financial strain led to an increase in both sleep disturbance and psychological distress, which both resulted in an increase in smoking. Surprisingly, an increase in socioeconomic status led to an increase in sleep disturbance, but this was a very small effect. The direct effect of sleep disturbance on smoking was not significant, but the specific direct effect of sleep disturbance on psychological distress was the largest of any pathway in the model. A difference in sleep disturbance of one standard deviation, holding constant socioeconomic status and financial strain, was associated with psychological distress that was 0.53 standard deviations higher. Out of the four mediating variables, psychological distress (0.171) had the largest total effect on smoking, followed by sleep disturbance (0.138), social cohesion (-0.100), and financial strain (0.052).

**Table 4:** Total, direct, and indirect standardized effects from socioeconomic status to current smoking status (3-levels).

	(1) Social Cohesion, financial strain		(2) Psychological distress sleep disturbance		(3) Social Cohesion, financial strain, psychological distress		(4) Social Cohesion, financial strain, psychological distress, sleep disturbance	
**Total (SES to smoking)**	-0.228	<0.001	-0.221	<0.001	-0.222	<0.001	-0.258	<0.001
**Total Indirect (SES to smoking)**	-0.099	<0.001	-0.052	<0.001	-0.116	<0.001	-0.087	<0.001
**Direct (SES to smoking)**	-0.129	<0.001	-0.169	0.029	-0.106	<0.001	-0.171	<0.001
**Specific indirect**								
SES to social cohesion to smoking	-0.042	0.001	–	–	-0.038	<0.001	-0.027	<0.001
SES to financial strain to smoking	-0.057	0.003	–	–	-0.043	<0.001		
SES to sleep disturbance to smoking	–	–	-0.005	<0.001			0.004	0.074
SES to psychological distress to smoking	–	–	-0.035	0.002	-0.017	<0.001	-0.036	<0.001
SES to sleep disturbance to psychological distress to smoking	–	–	-0.012	0.001	–	–	0.007	<0.001
SES to social cohesion to psychological distress to smoking	–	–	–	–	-0.005	<0.001	–	–
SES to financial strain to psychological distress to smoking	–	–	–	–	-0.014	<0.001	-0.011	<0.001
SES to s. cohesion to sleep disturbance to smoking	–	–	–	–	–	–	-0.003	0.057
SES to financial strain to sleep disturbance to smoking	–	–	–	–	–	–	-0.005	0.056
SES to social cohesion to sleep disturbance to psychological distress to smoking	–	–	–	–	–	–	-0.006	<0.001
SES to financial strain to sleep disturbance to psychological distress to smoking	–	–	–	–	–	–	-0.010	0.002
**Total Effects**								
Total effects of social cohesion	-0.119	–	–	–	-0.122	–	-0.100	–
Total effects of financial strain	0.180	–	–	–	0.183	–	0.052	–
Total effects of sleep disturbance	–	–	0.086	–	–	–	0.138	–
Total effects of psychological distress	–	–	0.125	–	0.112	–	0.171	–

## Discussion

We observed significant mediation between socioeconomic status and smoking through each of four latent variables: social cohesion, financial strain, sleep disturbance, and psychological distress. Although the direct influence of social cohesion on smoking was small, the overall influence of social cohesion in decreasing the probability of smoking was amplified by its effect on decreasing sleep disturbance. Sleep disturbance had no significant independent influence on smoking, but had a large total influence by increasing psychological distress which then increased the probability of smoking.

These findings highlight the significant correlation and role of sleep disturbance and psychological distress in mediating the inverse association between socioeconomic status and smoking. Of all the relationships tested in the model, the largest influence was a positive effect of sleep disturbance on psychological distress. This in turn led to a significant overall effect of sleep disturbance on smoking. In models that explored its mediating role, sleep disturbance had a large influence on smoking, particularly among females and the younger age group. Evidence suggests that improving sleep through the use of cognitive behavior therapy for chronic insomnia may improve psychological endpoints related to affective and anxiety disorders [75–77]. Future research could fruitfully explore how treatment of sleep disturbance could not only ameliorate psychological distress but also reduce smoking [78].

These conceptual models clearly did not incorporate all mediators between socioeconomic status and smoking behaviors, as evidenced by the remaining and influential significant direct effects. Other factors potentially mediate the relationship, such as parental and peer smoking behaviors, tobacco industry marketing, health concerns, and self-efficacy to quit [6,7,79].

Future studies could address some of this study’s limitations. First, because data used in this study were cross-sectional, we cannot account for the timing of the exposures, mediating variables, and outcome. Our interpretation assumes that current socioeconomic status and mediating variables are stable, and have effects on current smoking status in the order. As with many epidemiological studies, we were limited to observational data and cannot interpret findings as definitive of mediation or causation. Second, we were limited to the variables and responses coded in the existing NHIS data source. Third, in order to test a recursive model, we had to decide on one direction of effects, although the relationship of some factors may be bi-directional. Psychological distress has been demonstrated to affect sleep quality [76,77]; however, our model assumed sleep disturbance had a direct effect on psychological distress in order to test the theory that the treatment of sleep could improve symptoms of psychological distress. Finally, even though our models fit the data reasonably well, it is possible that other models or configurations would have fit the data equally well or better. Despite these limitations, we used this as an exploratory analysis to generate hypotheses and to explore the use of SEM in epidemiological studies.[18,80]

Alongside these limitations, this study had several strengths, so that its findings are an important contribution to understanding the mechanisms that link socioeconomic status and smoking. This study was conducted using a very large sample representative of the U.S. non-institutionalized adult population; therefore, the study was not limited by a small sample size as in other SEM studies. SEM allowed us to test multiple relationships simultaneously within a conceptual model, which is important in a research area where several mediating variables are suspected to have complex intercorrelations. Finally, we believe that this is the first study to examine sleep disturbance and a direct measure of psychological distress as potential mediators between socioeconomic status and smoking.

A better understanding of the complex relationship between socioeconomic status and smoking is critical as we continue to reduce the burden of tobacco and eliminate health disparities related to smoking. This study examined multiple mediators that may serve as potential areas to intervene. Further research will identify variables other than the ones studied here that also contribute to the higher smoking prevalence observed among populations of low socioeconomic status.
